# P53 in lung vascular barrier dysfunction

**DOI:** 10.1530/VB-20-0004

**Published:** 2020-04-28

**Authors:** Nektarios Barabutis

**Affiliations:** 1 School of Basic Pharmaceutical and Toxicological Sciences, College of Pharmacy, University of Louisiana Monroe, Monroe, Louisiana, USA

**Keywords:** inflammation, growth hormone, Hsp90, acute lung injury, acute respiratory distress syndrome

## Abstract

Endothelial barrier dysfunction is the hallmark of inflammatory lung disease, including Acute Lung Injury and Acute Respiratory Distress Syndrome. The purpose of the present editorial is to emphasize on recent advances in the corresponding field, as it relates to P53. This tumor suppressor protein has been shown to enhance the vascular barrier integrity via distinct molecular pathways. Further, it mediates the beneficial effects of heat shock protein 90 inhibitors and growth hormone releasing hormone antagonists in the lung microvasculature.

Lung endothelial barrier dysfunction is the hallmark of Acute Respiratory Distress Syndrome (ARDS), the most severe form of Acute Lung Injury. In ARDS, pulmonary interstitial and alveolar edema augments the intra-pulmonary shunt and reduces the functional lung size due to alveolar damage. Systemic inflammation frequently occurs, and ARDS patients are subjected to the fatal complications of sepsis-induced multiple organ failure. The incidence of ARDS in the United States ranges from 64.2 to 78.9 cases/100,000 person-years, and unfortunately, in severe cases, the mortality rate raises up to 45%. Hence, the development of new therapeutic approaches toward ARDS is considered an urgent need (1).

Intense efforts are oriented toward the delineation of the mechanisms involved in the regulation of lung endothelial permeability. The identification of ‘key players’ in this function may lead to the discovery of new therapeutics interventions to enhance vascular barrier function. Such beneficial outcomes will alleviate the devastating consequences of ARDS and will enrich the armamentarium of therapies against inflammatory lung disease. The ARDS survivors, in their majority, experience lasting damage in their lungs and frequently present symptoms of anxiety, depression and post-traumatic stress disorder (2).

Wild type P53 is a tumor suppressor protein, which senses cellular threats and initiates cellular responses. This transcription factor has been associated with strong anti-inflammatory activities, partially due to the fact that both conditions are closely interrelated. Chronic inflammation promotes carcinogenesis and malignancies prevail in inflammatory sites (3). To investigate the role of the endothelium defender (4) in the integrity of the lung vasculature, we employed primary human lung microvascular endothelial cells, transfected with small interfering (si) RNA for P53. By measuring the transendothelial resistance (TEER) of the microvascular monolayers, we revealed the crucial role of P53 in the integrity of the pulmonary microvasculature. The TEER values of the lung cells that were silenced for P53 were lower than the corresponding control groups, indicating a weakened barrier function. Moreover, the si-RNA inflicted cells were more vulnerable to Lipopolysaccharides (LPS) compared to the controls (5). On the other hand, microvascular cells overexpressing P53 due to pharmacological P53 inducers (e.g. Nutlin), or lung cells from super-P53 mice (mutants overexpressing P53), were more resilient to the LPS challenge than the corresponding control groups (6). Direct measurements of the reactive oxygen species (ROS) by 2,7-Dichlorodihydrofluorescein diacetate in lung cells revealed that P53 enhances the endothelial barrier function by reducing the ROS levels. P53 suppression resulted to an increased reactive oxygen species production, associated with reduced TEER values, indicating vascular dysfunction (7).

Anti-inflammatory agents, initially developed to fight malignancies, delivered promising results against the LPS-induced lung injury, both *in vivo* and *in vitro*. Heat shock protein 90 (Hsp90) inhibitors are agents that block the maturation and activation of Hsp90 clients-inflammatory mediators (i.e. ERK1/2 kinases, JAK2/STAT3). They exert their protective activities in the vasculature via P53 induction and by suppressing the LPS-induced P53 phosphorylation (degradation) (8). Another class of advanced anti-cancer agents, namely the growth hormone releasing hormone (GHRH) antagonists, were shown to enhance lung endothelial barrier function via P53 induction, RhoA suppression and cofilin deactivation (9). GHRH regulates the release of growth hormone from the anterior pituitary gland by activating the corresponding full length GHRH receptor. Splice variants of that receptor are expressed in extra hypothalamic tissues, including lung microvascular endothelial cells (3).

Since both Hsp90 and P53 have been associated with the unfolded protein response (UPR), we are currently investigating the outcomes of its activation in the lungs. This element is a molecular machinery developed to protect the cells against potential threats. However, when the inflicted damage is irreversible, UPR will initiate cell death. To do so, it employs three sensors, namely the protein kinase RNA-like ER kinase, the activating transcription factor 6 and the inositol-requiring enzyme-1α. It was recently shown that Hsp90 inhibitors induce UPR activation and that GHRH antagonists elevate the endoplasmic reticulum stress (10). Hence, an efficient therapeutic approach toward ARDS may arise by the development of novel agents that are able to stochastically induce those UPR branches that are involved in the repair of the lung endothelium. A new and potentially more advanced generation of GHRH antagonists and Hsp90 inhibitors may serve this purpose.

## Declaration of interest

The author declares that there is no conflict of interest that could be perceived as prejudicing the impartiality of this editorial.

## Funding

Dr Barabutis’s research is supported by the R&D, Research Competitiveness Subprogram (RCS) of the Louisiana Board of Regentshttp://dx.doi.org/10.13039/100006952 through the Board of Regents Support Fund (LEQSF(2019-22)-RD-A-26).
